# Improved volume variable cluster model method for crystal-lattice optimization: effect on isotope fractionation factor

**DOI:** 10.1186/s12932-022-00078-6

**Published:** 2022-05-22

**Authors:** Yan-Fang Wang, Xin-Yue Ji, Le-Cai Xing, Peng-Dong Wang, Jian Liu, Tian-Di Zhang, Hao-Nan Zhao, Hong-Tao He

**Affiliations:** 1 School of Earth Science and Engineering, Hebei University of Engineering, Handan, 056038 China; 2 Key Laboratory of Resource Survey and Research of Hebei Province, Hebei University of Engineering, Handan, 056038 China

**Keywords:** Molecular cluster, VVCM, Geometric optimization, Relative volume change, Isotopic equilibrium fractionation factor

## Abstract

The isotopic fractionation factor and element partition coefficient can be calculated only after the geometric optimization of the molecular clusters is completed. Optimization directly affects the accuracy of some parameters, such as the average bond length, molecular volume, harmonic vibrational frequency, and other thermodynamic parameters. Here, we used the improved volume variable cluster model (VVCM) method to optimize the molecular clusters of a typical oxide, quartz. We documented the average bond length and relative volume change. Finally, we extracted the harmonic vibrational frequencies and calculated the equilibrium fractionation factor of the silicon and oxygen isotopes. Given its performance in geometrical optimization and isotope fractionation factor calculation, we further applied the improved VVCM method to calculate isotope equilibrium fractionation factors of Cd and Zn between the hydroxide (Zn–Al layered double hydroxide), carbonate (cadmium-containing calcite) and their aqueous solutions under superficial conditions. We summarized a detailed procedure and used it to re-evaluate published theoretical results for cadmium-containing hydroxyapatite, emphasizing the relative volume change for all clusters and confirming the optimal point charge arrangement (PCA). The results showed that the average bond length and isotope fractionation factor are consistent with those published in previous studies, and the relative volume changes are considerably lower than the results calculated using the periodic boundary method. Specifically, the average Si–O bond length of quartz was 1.63 Å, and the relative volume change of quartz centered on silicon atoms was  − 0.39%. The average Zn–O bond length in the Zn–Al-layered double hydroxide was 2.10 Å, with a relative volume change of 1.96%. Cadmium-containing calcite had an average Cd–O bond length of 2.28 Å, with a relative volume change of 0.45%. At 298 K, the equilibrium fractionation factors between quartz, Zn–Al-layered double hydroxide, cadmium-containing calcite, and their corresponding aqueous solutions were $\Delta ^{30/28} {\text{Si}}_{{{\text{Qtz-H}}_{4} {\text{SiO}}_{4} }}  = 2.20{\permil}$, $\Delta^{18/16} {\text{O}}_{ {\text{Qtz}}{-} ( {\text{H}}_{2} {\text{O}} )_{\text{n}}}  = 36.05{\permil}$, $\Delta^{66/64} {\text{Zn}}_{ {\text{Zn}} {-} {\text{Al LDH-Zn}} ( {\text{H}}_{2} {\text{O}}  )_{\text{n}}^{2+}} = 1.12{\permil}$ and $\Delta^{114/110}  {\text{Cd}}_{ {\text{(Cd--Cal)-Cd}}   ( {\text{H}}_{2} {\text{O}} )_ {\text{n}}^{2 +} } = - 0.26{\permil}$ respectively. These results strongly support the reliability of the improved VVCM method for geometric optimization of molecular clusters.

**Supplementary Information:**

The online version contains supplementary material available at 10.1186/s12932-022-00078-6.

## Introduction

In nature, the isotopic composition of an element varies with rock type, source, and age, especially those determined by high-resolution mass spectroscopy since the 2000s. This has considerably expanded the usefulness of isotopic tools, which have been widely used to study processes occurring in terrestrial, atmospheric, and aquatic environments [1–7]. Specifically, the composition of isotopes can be used for geological temperature measurement and geological dating [8–11], as well as to determine the genesis of ore deposits [12, 13]. Moreover, several important geological events are reflected by the sudden change in isotope composition, such as the largest mass extinction at the Permian–Triassic boundary, revealed by seawater δ^7^Li and δ^114^Cd content [14, 15]. Therefore, isotopes also play an important role in tracing paleoclimatic variations [16]. Although isotopes have been widely studied in various fields of geochemistry, the magnitude of isotope fractionation is unknown. This has substantially hindered the development of stable isotope geochemistry. However, the magnitude of isotope fractionation is a key parameter for establishing an isotope evolution model. At present, three methods are used to obtain the isotope equilibrium fractionation factor: experimental determination, empirical estimation, and theoretical calculation. However, laboratory experiments are often conducted under conditions that deviate from natural conditions, such as elevated background concentrations, high temperatures, and short durations. Hence, extrapolation from the laboratory-derived results to geological settings requires further evaluation. The accuracy of an empirical estimation is dependent on the parameters adopted in the calculations, which may result in a large error in the obtained fractionation factor. Therefore, theoretical calculations have become a useful tool for obtaining precise isotope equilibrium fractionation factors. With the rapid increase in storage and computing efficiency in recent decades, supercomputers are now being used to model increasingly large systems of up to tens of thousands of atoms. Computational simulations, particularly quantum chemical simulations, consider various chemical interactions, mimicking key reaction steps. The accuracy of the calculated results is equivalent to that of the experimental analyses. Simulations have proven particularly useful, especially when sampling and/or experimental constraints are difficult to obtain. Among the various simulation methods, first-principles calculations based on density functional theory (DFT) have been widely used to calculate isotopic fractionation factors, element partition coefficients, and vibrational spectra. However, obtaining stable geometrical configurations is a prerequisite for calculating these parameters. The harmonic vibrational frequency required to calculate the isotopic fractionation factor is a second-order partial derivative of the electronic energy of the system with respect to its atomic coordinates.

Research on solid earth science involves the geometric optimization of solid structures. The periodic boundary method is the most commonly used method for simulating solids. Mineral cells are used to construct periodic systems. In general, supercells are required to ensure that the constructed system is comparable to the real mineral lattice, but the total number of unit cells is limited by computational power. In addition, improper handling of atoms at the boundary of the supercells may considerably affect the accuracy of the calculated results. Given the shortcomings of the periodic boundary method, Rustad and Dixon [17], Rustad and Yin [18], and Rustad et al. [19, 20] introduced an embedded cluster model with a Pauling bond strength (PBS)-conserving termination of the atoms in their rigid shell. In this model, a large molecular cluster with three shells is employed to represent the minerals. A small core and a second shell are optimized at high and low levels of theory and basis sets, respectively. The third shell is fixed at the measured lattice positions. Chemical bonds entering the third shell are clipped and replaced by “Pauling bond strength-conserving quasi-atoms” along the clipped bonds, which make the core charge of each quasi-atom equal to the Pauling bond valence, that is, the charge of the removed cation divided by its coordination number. The distance between the quasi-atom and the third shell is maintained at 1 Å. Finally, the frequency calculations are performed only for the small core. As an alternative, the volume variable cluster model (VVCM) method was proposed by Liu [21], which was developed during the optimization of silicates, carbonates, oxides, hydroxides, and sulfides [22–28]. In contrast to the embedded cluster model, in the VVCM model all atoms of the cluster are geometrically optimized, and all or partial atoms are involved in the calculations of the vibrational frequency according to the computational cost. The total number of background point charges amounts to several hundreds, which is much higher than the number of quasi-atoms in the embedded cluster model. This ensures the coverage of point charges on the entire molecular cluster. Additionally, the positions of the background point charges are adjusted during the construction of the initial molecular clusters. More importantly, geometric optimization and harmonic vibrational frequency calculations can be performed for both periodic systems (minerals) and nonperiodic systems (solutions) with identical exchange–correlation functionals and basis sets. Although the main two limitations of the molecular cluster method are the same as those of the periodic boundary method (the size of the system and the treatment of atoms on the boundary) and its representation of the periodic mineral lattice is not as accurate as that of the periodic boundary method, it is able to precisely treat the local configuration around the atom of interest, which is the dominant factor controlling isotope fractionation. In recent years, static quantum chemistry calculations based on molecular clusters have accurately calculated the stable isotope equilibrium fractionation factors [22–32]. The calculated results are in agreement with previous experimental and theoretical calculations. However, the critical question is to treat the local configuration around the atom of interest. In previous studies, a detailed procedure to obtain an optimized local configuration was absent. In addition, evaluation proxies for the optimized local configuration are not well-established. The energy of a solid (E) is a function of the molecular volume (V) [33], total number of atoms, and interactions among the atoms. Additionally, the relationship between energy and volume is linear and/or nonlinear [34]. For example, the nonlinear four-parameter Vinet equation of state [35, 36] can be expressed as follows:

1 $${\text{E}}({\text{V}}) = {\text{a}} - \frac{{4{\text{B}}_{0} {\text{V}}_{0} }}{{\left( {{\text{B}}_{0}^{\prime } - 1}\right)^{2} }}\left\{ {1 - \frac{3}{2}\left( {{\text{B}}_{0}^{\prime } - 1} \right)\left[ {1 - \left( {1 + \frac{{\Delta {\text{V}}}}{{{\text{V}}_{0} }}} \right)^{{{1 \mathord{\left/ {\vphantom {1 3}} \right. \kern-\nulldelimiterspace} 3}}} } \right]} \right\} \times \exp \left\{ {\frac{3}{2}\left( {{\text{B}}_{0}^{\prime } - 1} \right)\left[ {1 - \left( {1 + \frac{{\Delta {\text{V}}}}{{{\text{V}}_{0} }}} \right)^{{{1 \mathord{\left/ {\vphantom {1 3}} \right. \kern-\nulldelimiterspace} 3}}} } \right]} \right\}$$

where the fitting parameter a = E_0_ + 4B_0_V_0_/(B_0_^’^-1)^2^, V_0_, B_0_, and B^’^_0_ represent the equilibrium volume, bulk modulus, and its first derivative with respect to pressure, respectively; and ΔV is the deviation from the equilibrium volume.

Given the role of molecular volume in the energy of the solid, the expansion or contraction of molecular clusters will influence the accuracy of harmonic vibrational frequencies and the isotope fractionation factor. However, the degree of expansion or contraction of molecular clusters was not considered in the previous molecular cluster modeling method.

In this study, we chose the typical oxide quartz (Qtz) present on the Earth’s surface [37, 38] as an example. By geometrically optimizing the Qtz molecular cluster, extracting its parameters (converged electronic energy, average bond length, and relative volume change), and calculating the equilibrium oxygen and silicon isotopic fractionation factors between molecular clusters and the aqueous solution, we aimed to verify the usefulness of the improved VVCM method after comparison of methods developed in previous studies. Based on the performance of the improved VVCM method for Qtz, we extended the method to the calculation of other nontraditional stable isotopes (Zn and/or Cd) between layered double hydroxide (Zn–Al LDH), carbonate (Cd–containing calcite, Cd–Cal), and their corresponding solutions. After rechecking previous theoretical results for cadmium-containing hydroxyapatite (Cd–HAp), emphasizing the relative volume change for all clusters, and confirming the optimal point charge arrangement (PCA), we constructed a detailed procedure to implement the improved VVCM method.

## Theory and methods

**Fig. 1:**
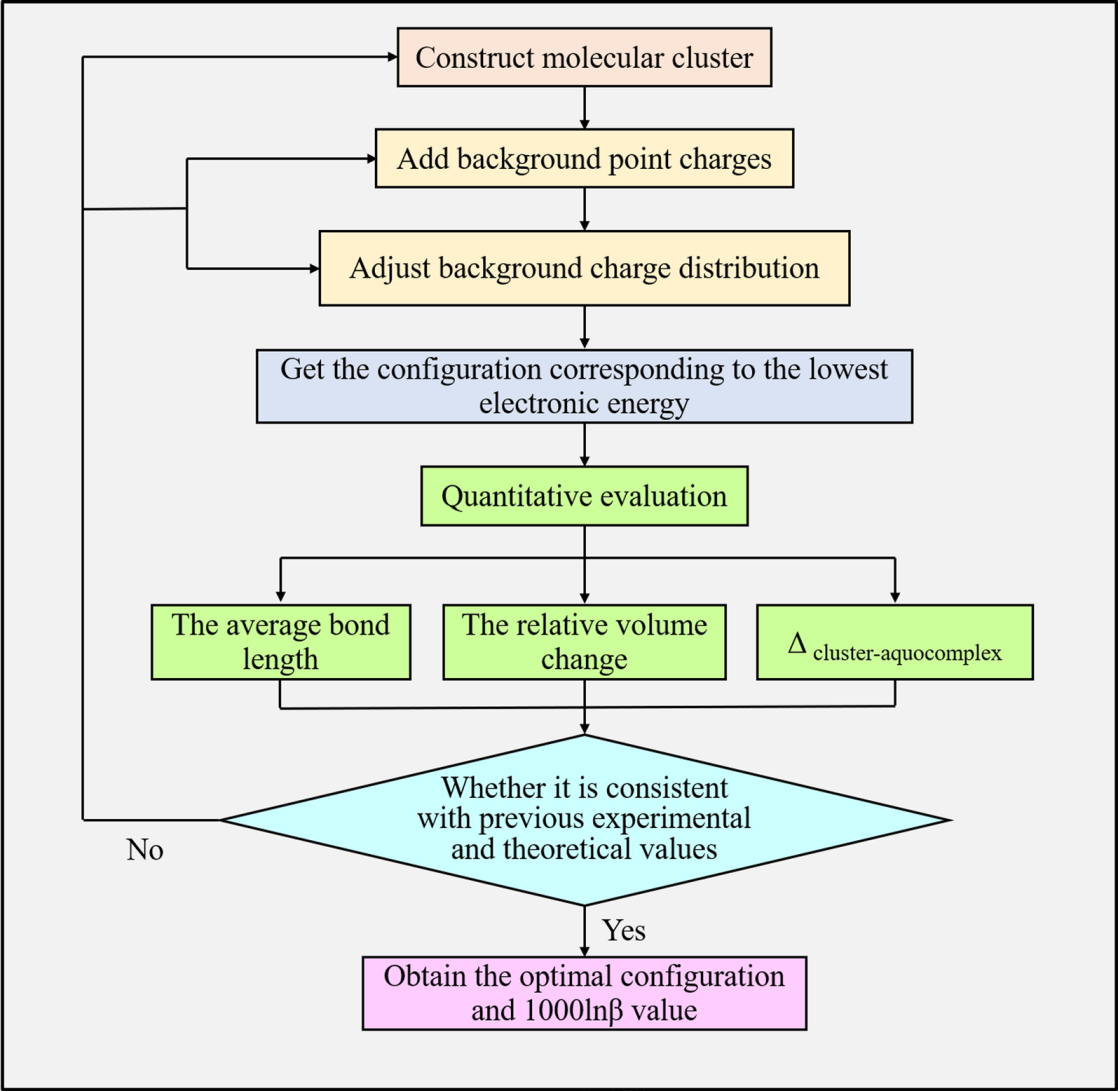
Flow chart of the improved VVCM method

To obtain an optimal geometric structure, we systematically used the optimization process based on the improved VVCM method, as shown in Fig. 1.

### Constructing molecular cluster and adding background point charges

We reconstructed the three-dimensional crystal lattices of the minerals from X-ray single-crystal diffraction or neutron diffraction data obtained from previous studies [39–43]. The N–N–N principle must be followed when cutting molecular clusters. This requires the atoms (nearest neighbor) to form chemical bonds with the central atom (atom of interest), and the atoms (the second neighbor atom) forming chemical bonds with the nearest neighbor must be retained. In specific cases, the size of the molecular clusters depends on the computational cost and accuracy. Generally, the outermost layer of a cluster is composed of anions or anionic groups, while the metal ions and/or protons connected to these anions or anionic groups are deleted. The hanging bonds are typically saturated with positive point charges to fix the outermost atoms and to simulate the electron-neutral environment of the crystal. Existence of free interlayer anions makes LDH different from others. The general formula of LDH is [M^2+^_1−x_ M^3+^_x_ (OH)_2_]^x+^(A^n−^)_x/n_·mH_2_O, where M^2+^ and M^3+^ represent divalent and trivalent metal cations, respectively; A^n−^ is an interlayer anion including CO_3_^2−^, Cl^−^, SO_4_^2−^, NO_3_^−^; x represents the molar ratio M^3+^/(M^2+^  + M^3+^); and m is the number of water molecules [44, 45]. In nature, the M^2+^/M^3+^ molar ratio is usually two [46]. Therefore, we focused on a typical Zn-containing LDH with a Zn:Al ratio of two. We constructed two kinds of molecular clusters for LDH without and with interlayer anions.

**Fig. 2:**
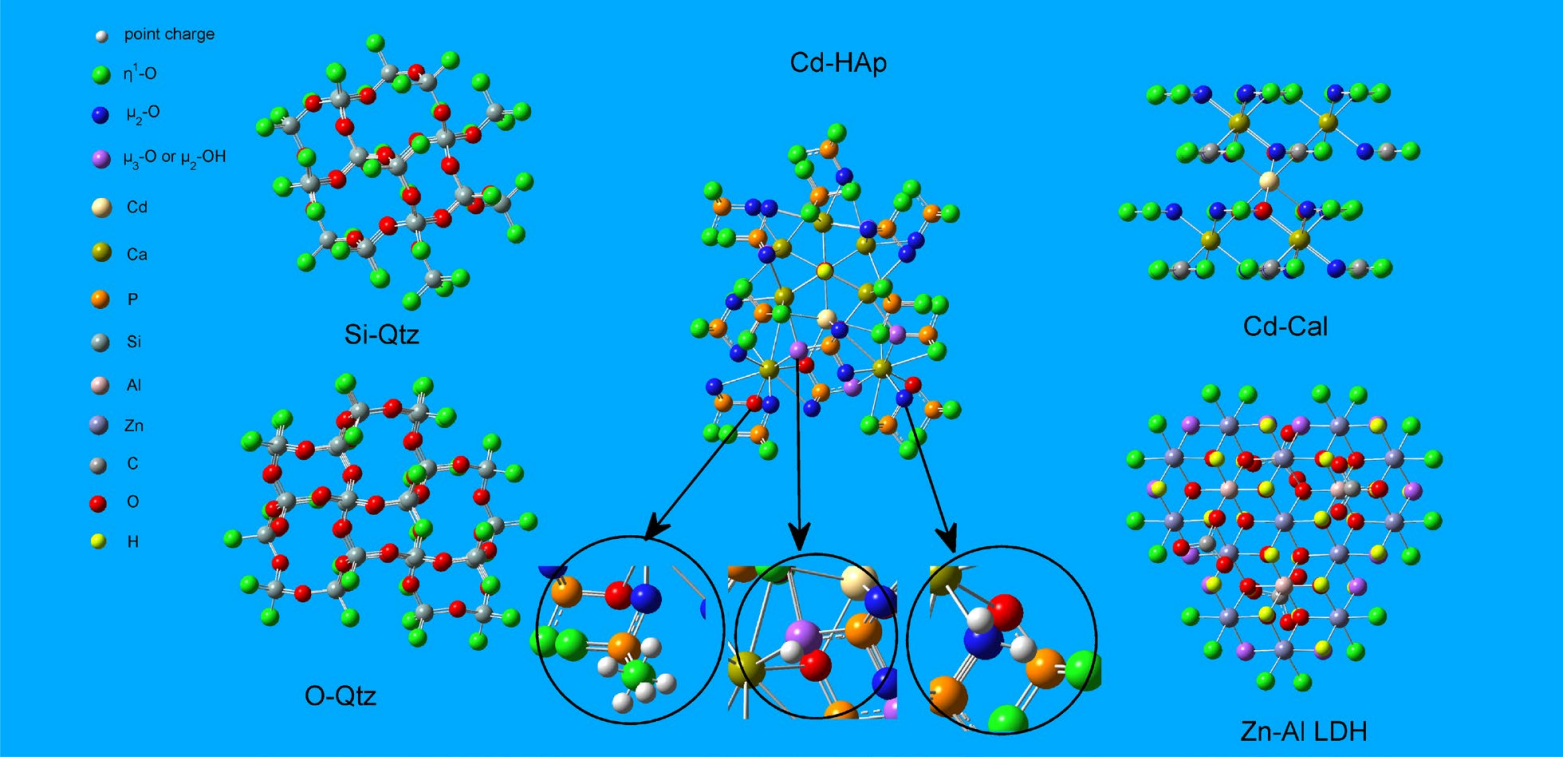
Schematic diagram of PCA

Oxygen atom can strongly attract the electron of hydrogen atom due to its strong electronegativity. It means that each hydrogen atom of a water molecule can accept a non-shared electron pair of the oxygen atom. Meanwhile, oxygen atom can contribute its two non-shared electron pairs to two hydrogen atoms. Therefore, water molecules can form 4 H bonds with each other in solution [47], which constructs a tetrahedron structure found in ice crystals (Additional file 1: Figure S1). Inspired by this observation, we used point charges and connected chemical bonds to encompass the terminal anions. Different terminal anions have various arrangements of positive point charges. In a previous study, three PCAs were proposed [25], and for the terminated oxygen (η^1^-O) capped by five positive point charges (5 ×), two positive point charges were used as two wings (2 ×) distributed on the bridge oxygen (µ_2_-O) or the terminated hydroxyl (η^1^-OH), and one positive point charge was set to the apex (1 ×) of the bridge oxygen or hydroxyl (µ_3_-O or µ_2_-OH) (Fig. 2). Considering η^1^-O as the predominant anion on the surface of the cluster, we added point charges in four different ways to evaluate their effect on the theoretical results. The one was to add one point charge in the direction of the deleted atoms [17–20]. In other three ways, three, four, and five point charges were added to η^1^-O, respectively. However, point charges were not fixed at the directions of chipped bonds. Alternatively, these point charges were averagely distributed in the space surrounding the η^1^-O. Obviously, η^1^-O is more tightly capped by five point charges than by three or four point charges (Additional file 1: Figure S1). Furthermore, we performed geometrical optimizations for the Qtz cluster considering 1 ×, 3 × , and 5 × PCAs (Table 1). Although the average Si–O bond length optimized in three PCAs was slightly larger than that in the experimental data (1.61 Å [39] and 1.62 Å [48]), the more accurate relative volume change (ΔV/V_0_) and $\Delta^{{{{30} \mathord{\left/ {\vphantom {{30} {28}}} \right. \kern-\nulldelimiterspace} {28}}}} {\text{Si}}_{{{\text{Qtz - H}}_{4} {\text{SiO}}_{4} }}$ indicated that 5 × was the best PCA for η^1^-O. Similarly, we safely capped µ_2_-O and µ_3_-O with two and one point charges, respectively, according to a previous study [25].

**Table 1:** Comparison of theoretical results for three optimal PCAs

Optimal PCA (pm)	Si–O bond length (Å)	ΔV/V_0_ (%)	$\Delta^{{{{30} \mathord{\left/ {\vphantom {{30} {28}}} \right. \kern-\nulldelimiterspace} {28}}}} {\text{Si}}_{{{\text{Qtz - H}}_{4} {\text{SiO}}_{4} }}$ (298 K, ‰)
1 × 94	1.630	2.03	0.78
3 × 111	1.629	1.00	1.76
5 × 116	1.625	− 0.39	2.02

Before optimizing the configuration, we set the charge quantity and distance of the point charges of the outermost oxygen atoms. The quantity of charge was equal to the amount of charge provided by the outermost anions or anionic groups of the cluster. Specifically, the total amount of point charge on each terminal anion was equal to the sum of the contributions of all “cut” metal ions and/or protons. The distance between the point charge and the outermost oxygen atom was set empirically. Upon adjustment of the distance between the point charge and outermost oxygen atoms, the electronic energy of the system also changed, and each atom of the inner part of the cluster relaxed under its three freedoms (X, Y, and Z dimensions). When the inner atoms amounted to N, 3 N freedoms were observed. The outermost layers of the Qtz clusters centered on silicon or oxygen were all η^1^-O (Fig. 2). In the Qtz cluster, the total electrical charge of the point charges on each η^1^-O was equal to 1/4 of that of the removed Si^4+^, that is, + 1 (Table 2). Therefore, the partial electrical charge of each point charge was set to + 1/5. In the Zn–Al LDH cluster, the two types of PCAs were 5 × and 1 × (Fig. 2). The total amount of charge on η^1^-O was equal to the total amount of charge contributed by the removed Zn^2+^, Al^3+^, and H atoms. Specifically, the total amount of charge was the summation of the charge contributed by one hexa-coordinated Zn^2+^ (+ 2/6), one hexa-coordinated Al^3+^ (+ 3/6), and one H atom (+ 1). Thus, the total amount of charge on η^1^-O was + 11/6. We set the electric quantity of each point charge to + 11/30. The total charge on µ_2_-OH was equal to the removed charge of one hexa-coordinated Al^3+^ (+ 3/6). The electric quantity of each point charge was + 1/2. Furthermore, for Cd–Cal, the two PCA modes were 5 × and 2 × (Fig. 2). The total charge on η^1^-O was equal to the total amount of charge of the two removed hexa-coordinated Ca^2+^ (2/6 + 2/6), so the amount of charge divided by each point charge was + (2/6 + 2/6)/5. The total amount of charge on μ_2_-O was + 2/6, and the electric quantity of each point charge was + (2/6)/2.

**Table 2:** PCA on the anion or anion group in the outermost layer of a molecular cluster

Cluster	Molecular formula	Total valence	1 ×		2 ×			5 ×		Net charge
			^a^1 ×	^b^1 ×	^a^2 ×	^b^2 ×	^c^2 ×	^a^5 ×	^b^5 ×	
Si–Qtz	Si_24_O_66_	− 36	–		–			180 × (4/4) × 1/5		0
O–Qtz	Si_29_O_77_	− 38	–		–			190 × (4/4) × 1/5		0
Zn–Al LDH	Zn_13_Al_3_O_60_C_4_H_30_	− 39	12 × 3/6		–			90 × (1 + 3/6 + 2/6) × 1/5		0
Cd–Cal	Ca_6_Cd(CO_3_)_26_	− 38	–		60 × 2/6 × 1/2			210 × (2/6 + 2/6) × 1/5		0
Cd–HAp	Ca_12_Cd(PO_4_)_30_(OH)_3_	− 67	2 × 2/7	10 × 2/9	24 × (2/9 + 2/9) × 1/2	16 × (2/7 + 2/7) × 1/2	40 × (2/7 + 2/9) × 1/2	150 × (2 × 2/9 + 2/7) × 1/5	140 × (2 × 2/7 + 2/9) × 1/5	0

The outermost layer of the Si–Qtz molecular cluster has 5 × point charges (180). The outermost layer of the O–Qtz molecular cluster has 5 × point charges (190). The outermost layer of the Zn–Al LDH molecular cluster has 1 × point charges (12) and 5 × point charges (90). The outermost layer of the Cd–Cal molecular cluster has 2 × point charges (60) and 5 × point charges (210). The outermost layer of the HAp molecular cluster has ^a^1 × (2), ^b^1 × (10), ^a^2 × (24), ^b^2 × (16), ^c^2 × (40), ^a^5 × (150), and ^b^5 × (140) point charges

Si–Qtz net charge (0) = total cluster valent (-36) + total point charge (+ 36). O–Qtz net charge (0) = total cluster valent (-38) + total point charge (+ 38). Zn–Al LDH net charge (0) = total cluster valent (-39) + total point charge (+ 39). Cd–Cal net charge (0) = total cluster valent (-38) + total charge of point charge (+ 38). Cd–HAp net charge (0) = total cluster valent (-67) + total charge of point charge (+ 67)

The configuration of Cd-containing hydroxyapatite is further complicated by the simultaneous existence of η^1^-O, µ_2_-O, and µ_3_-O in its outermost layer (Fig. 2). Two ways can be used to set the electric quantity for each point charge in the 5 × mode. One fraction of η^1^-O obtains the total charge of two nine-coordinated Ca^2+^ and one seven-coordinated Ca^2+^ (2 × 2/9 + 2/7), setting the electric quantity of each point charge to + (2 × 2/9 + 2/7)/5. The other fraction of η^1^-O obtains the total charge (2 × 2/7 + 2/9) of two seven-coordinated Ca^2+^ and one nine-coordinated Ca^2+^, with the electric quantity of each point charge set to + (2 × 2/7 + 2/9)/5. The three ways to set the electric quantity for each point charge when adding two points of charge are as follows: (1) the µ_2_-O obtains the total charge of two nine-coordinated Ca^2+^ (2/9 + 2/9) and the electric quantity of each point is set to + (2/9 + 2/9)/2; (2) the µ_2_-O obtains the total charge of two seven-coordinated Ca^2+^ (2/7 + 2/7) and the electric quantity of each point is set to + (2/7 + 2/7)/2; and (3) the µ_2_-O obtains a total charge of a seven-coordinated Ca^2+^ and a nine-coordinated Ca^2+^ (2/7 + 2/9) and the electric quantity of each point is set to + (2/7 + 2/9)/2. The two types of electric quantities for each point charge in the 1 × modes are as follows: the electric quantity of each point charge on μ_3_-O is set to + 2/7 when the seven-coordinated Ca^2+^ is removed; and the point charge on μ_3_-O obtains a charge of + 2/9 when the nine-coordinated Ca^2+^ is removed.

### Molecular cluster optimization

We systematically adjusted the distance between the point charge and outermost oxygen atom to change the distribution of the background charge and to change the molecular cluster volume and geometry.

### Qtz

**Fig. 3:**
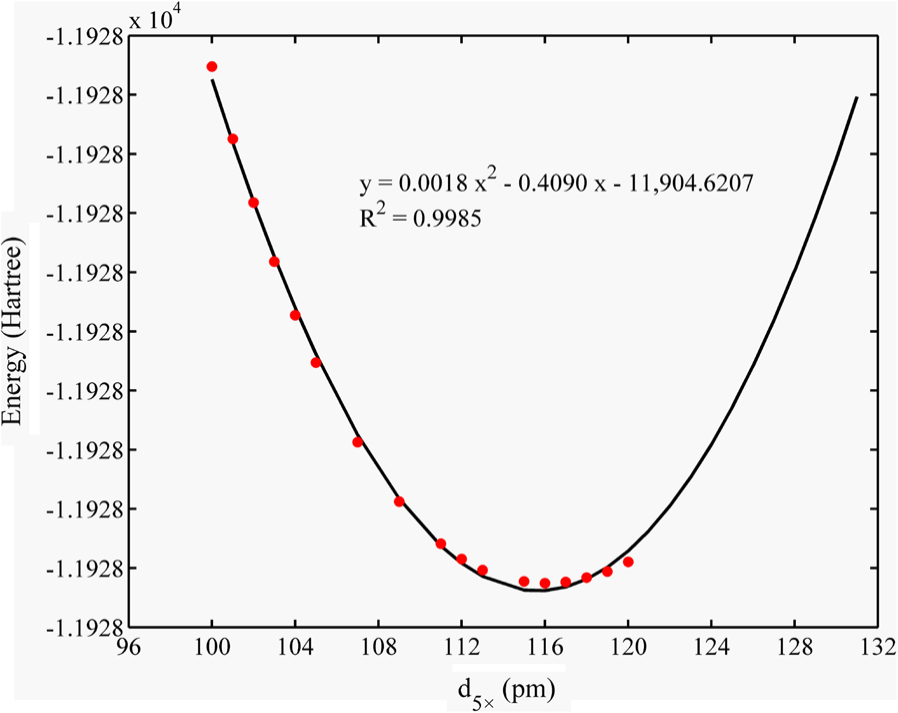
Electronic energy of the Si–Qtz system varies with d_5×_

Only one PCA exists in the Qtz molecular cluster, that is, 5 × PCA. Here, we considered the Qtz molecular cluster centered on a silicon atom as an example to provide a detailed description. According to previous studies, the range of distances between the 5 × point charge and η^1^-O (d_5×_) is approximately 100–120 pm [22]. Thus, we adjusted d_5×_ to 100 pm. Each time, we placed the point charge with two sets of distances, with a difference of only 1 pm, for instance, 100 and 101 pm. We determined the direction of further distance adjustment by determining the lower electronic energy (corresponding to 101 pm) in the two groups. Therefore, increasing the distance between the 5 × point charge and η^1^-O decreased the electronic energy of the cluster (Fig. 3). Subsequently, we set the point charge at two other distances (119 and 120 pm) and compared their electronic energies. Assuming that the electronic energy value of each configuration obeys a parabolic (concave upward) distribution, we determined that the point charge distance of the lowest electronic energy configuration should be between 101 and 119 pm. By further comparing the electronic energies of the system at medium distances of 111 and 112 pm, we further constrained the position of the parabola vertex within a narrower range (112–119 pm). Finally, we geometrically optimized all configurations within a small range to determine the optimal point charge distance corresponding to the lowest electronic energy value.

**Fig. 4:**
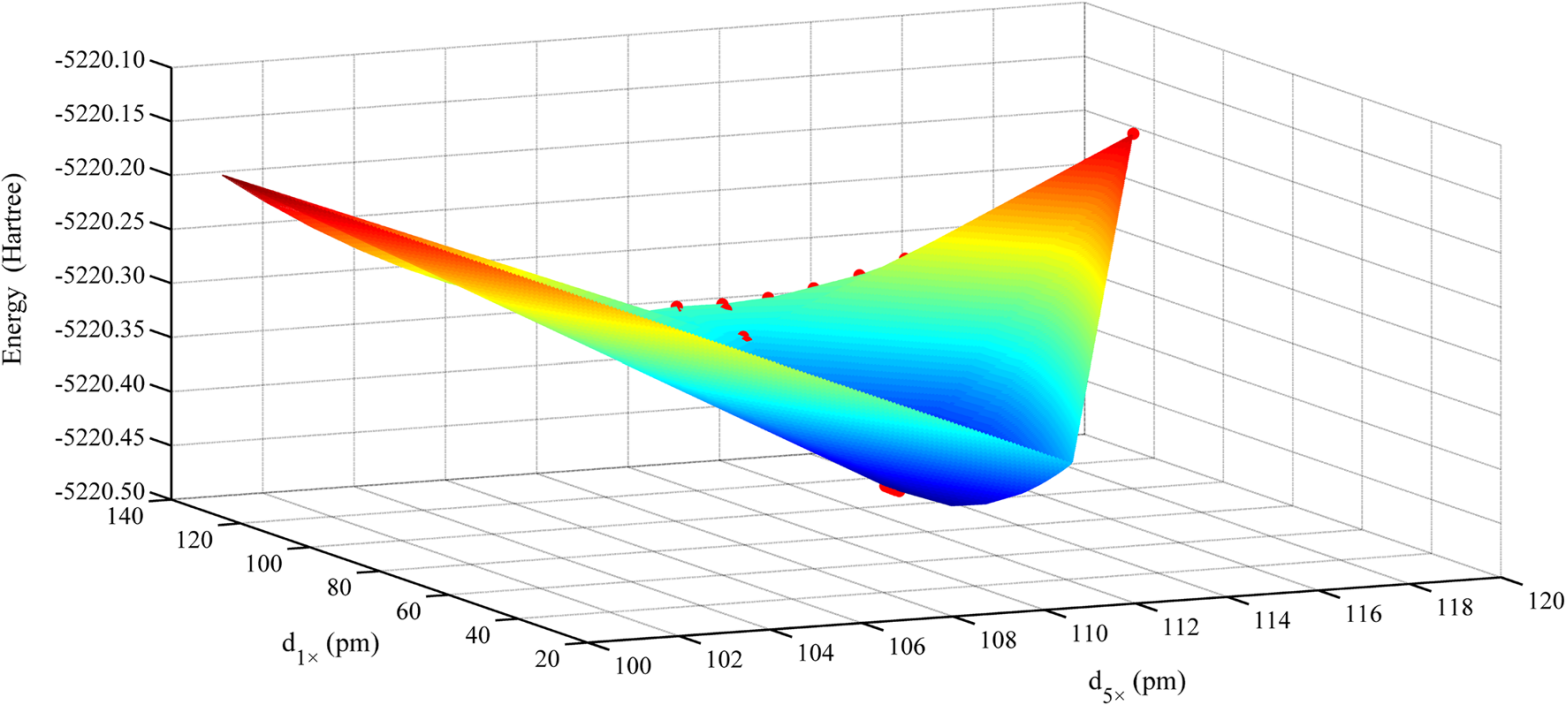
Electronic energy of the Zn–Al LDH system varies with d_5×_ and d_1×_

**Fig. 5:**
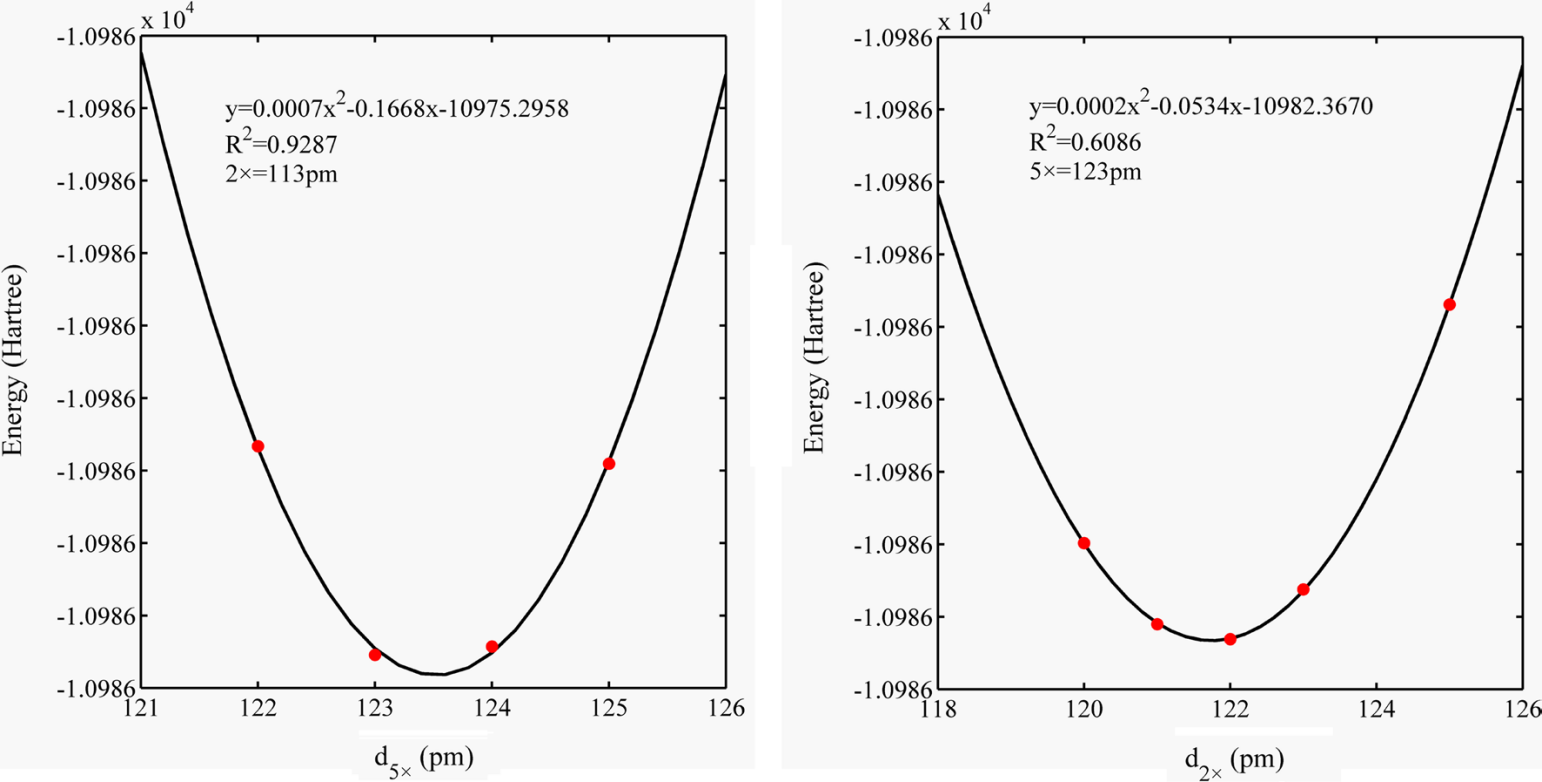
Electronic energy of the Cd–Cal system varies with d_5×_ and d_2×_

### Zn–Al LDH, Cd–Cal, and Cd–HAp

We used the optimal point charge distance search method, similar to that used for Qtz, for evaluating the Zn–Al LDH, Cd–Cal, and Cd–HAp molecular clusters. In the case of the coexistence of multiple PCA modes, we determined the most suitable distance for each PCA mode. Specifically, we arbitrarily fixed the distance between the 1 × point charge and μ_2_-OH or μ_3_-O (d_1×_) and/or the distance between the 2 × point charge and μ_2_-O (d_2×_). Subsequently, we successively adjusted d_5×_ until we determined the optimal value. For the Zn–Al LDH and Cd–Cal molecular clusters, we determined the optimal d_1×_ or d_2×_ after fixing d_5×._ However, confirming the most suitable distance d_2×_ (d_1×_) in the Cd–HAp molecular cluster requires d_1×_ (d_2×_) and d_5×_ to be both fixed.

In this study, we used the exchange–correlation functional B3LYP for Qtz and Zn–Al LDH, and BP86 for Cd–Cal and Cd–HAp. We used the all-electronic basis set 6–311G (d) [49, 50] to describe the wavefunction of the Qtz molecular cluster and a mixed basis set to describe the wavefunction of other systems: LanL2DZ for Zn [51–53]; LanL2MB for Cd [52]; 6–311 + G (d, p) for Al, O, and H in Zn–Al LDH; and 6–311G (d) for C, Ca, P, O, and H in Cd–Cal and Cd–HAp. We also performed harmonic vibrational frequency analyses to check whether there was an imaginary frequency and to ensure that the most stable configuration corresponded to at least the local minima on the potential electronic energy surface. We calculated electronic energy and performed geometrical optimization and harmonic vibrational frequency analyses using Gaussian09 code [54].

### Relative volume change

We calculated the relative volume change (ΔV/V_0_) to quantify the expansion or contraction of the molecular clusters during geometric optimization. By comparing the relative volume change (relative to the volume of the unoptimized molecular cluster) and the system electronic energy, we screened the configuration with little volume change with respect to the natural mineral lattice and the lowest electronic energy. Only this method could ensure that the simulation matched the real scenario to the largest extent. Because a single molecular cluster is not a unit cell or supercell of a mineral, accurately calculating the molecular cluster volume using cell parameters is impossible. The wavefunction or electronic density of a molecule is a function of space points. Moreover, the Monte Carlo method can be used to calculate the molecular cluster volume (molecular van der Waals volume), which is the space enclosed by an isosurface with an electron density of x e/Bohr^3^, where x represents the electron density value of the isosurface. We set up a rectangular box with volume V for molecular volume integration. Subsequently, we placed many random points (m) in the box for sampling and tested the electron densities at these space points to determine if they were larger than x e/Bohr^3^. Finally, we counted the number of points (n) with electron densities larger than x e/Bohr^3^, for which the van der Waals volume of the molecule was n/m*V [55, 56]. Bader et al. [57] proposed a more accurate definition of the van der Waals volume, stating that if a molecule is in the gas phase, an isosurface with an electron density of 0.001 e/Bohr^3^ is considered a van der Waals surface. Such surfaces typically contain more than 98% the electron density of a molecule. For molecules in a condensed state, intermolecular extrusion and various forms of interaction cause the van der Waals surface to penetrate. Using an isosurface with an electron density of 0.002 e/Bohr^3^ as the van der Waals surface is generally recommended. Among the molecular clusters constructed in this study, Cd–HAp had the largest number of atoms (no more than 169 atoms). Hence, we treated the molecular clusters as small molecules in the gas phase and used an isosurface with an electron density of 0.001 e/Bohr^3^ as the van der Waals surface of the molecular clusters. Cd–HAp was studied by He et al. [25], but they did not select the optimal PCA, by strictly adhering to our procedure. We rechecked the optimal PCA for this mineral, emphasizing the relative volume changes for all clusters.

Calculating the van der Waals volume requires the molecular wavefunctions. We obtained the wavefunction files using Gaussian09 code [54]. By inputting i, x, and k values, we calculated the volume using the Multiwfn program [58], where i is a proxy for the number of random points distributed in the box and k is the space reserved by the box around the molecule. Alternatively, the shortest distance between the outermost atom and the boundary of the box is k times the van der Waals radius of the atom. For specific calculations, we distributed 100 × 2^i^ random points in a box and set the value of k to 1.7. We used an isosurface with an electron density of 0.001 e/Bohr^3^ to envelop the molecule. Normally, to obtain a more accurate molecular van der Waals volume, the density of random points in the box must not be less than 2000 points/Bohr^3^. We used the following formula for these calculations:

2 $${{\Delta {\text{V}}} \mathord{\left/ {\vphantom {{\Delta {\text{V}}} {{\text{V}}_{0} }}} \right. \kern-\nulldelimiterspace} {{\text{V}}_{0} }} = \frac{{{\text{V}}_{{{\text{opt}}}} - {\text{V}}_{0} }}{{{\text{V}}_{0} }} \times 100\%$$

where V_opt_ denotes the optimized volume and V_0_ is the unoptimized volume. A positive ΔV/V_0_ indicates expansion of the molecular cluster, whereas a negative ΔV/V_0_ suggests contraction of the molecular cluster.

### Isotopic equilibrium fractionation factor

The Bigeleisen–Mayer equation and Urey model [59, 60] provide the equilibrium constants for the isotopic exchange reaction, laying the foundation for the theory and calculation of stable isotope geochemistry. Consider the following isotope exchange reaction as an example:

3 $${\text{AX}}^{\prime} + {\text{X}} = {\text{AX}} + {\text{X}}^{\prime}$$

where AX and AXʹ represent compounds with heavy and light isotopes, respectively; X and Xʹ denote the ideal single-atom gas with heavy and light isotopes, respectively. The reaction equilibrium constant K can be obtained as follows from the ratio of the partition functions of the products and reactants:

4 $${\text{K}} = \frac{{{\text{Q}}_{trans}^{{{\text{AX}}}} {\text{Q}}_{rot}^{{{\text{AX}}}} Q_{vib}^{{{\text{AX}}}} Q_{elec}^{{{\text{AX}}}} }}{{{\text{Q}}_{{{\text{trans}}}}^{{{\text{AX}}}^{\prime}} {\text{Q}}_{{{\text{rot}}}}^{{AX}}}^{\prime} Q_{{{\text{vib}}}}^{{{\text{AX}}}^{\prime}} Q_{{{\text{elec}}}}^{{AX}^{\prime}}}/\frac{{{\text{Q}}_{{{\text{trans}}}}^{{\text{X}}} {\text{Q}}_{{{\text{elec}}}}^{{\text{X}}}}}{{Q_{{{\text{trans}}}}^{{X}^{\prime}}}Q_{{{\text{elec}}}}^{{X}^{\prime}}}$$

where Q_trans_ is the transitional partition function, Q_rot_ is the rotational partition function, Q_vib_ is the vibrational partition function, and Q_elec_ is the electronic partition function [61]. For light elements, the difference in the ground-state electronic energy of the compound before and after isotope substitution is indistinguishable, so Q_elec_ can be ignored. The translational, rotational, and vibrational partition functions can be respectively written as follows:

5 $${\text{Q}}_{{{\text{trans}}}} = {\text{V}}\left( {\frac{{2\uppi {\text{MkT}}}}{{{\text{h}}^{2} }}} \right)^{3/2}$$

6 $${\text{Q}}_{{{\text{rot}}}} = \frac{{\uppi ^{{{1 \mathord{\left/ {\vphantom {1 2}} \right. \kern-\nulldelimiterspace} 2}}} \left( {8\uppi ^{2} {\text{kT}}} \right)^{{{3 \mathord{\left/ {\vphantom {3 2}} \right. \kern-\nulldelimiterspace} 2}}} \left( {{\text{I}}_{{\text{A}}} {\text{I}}_{{\text{B}}} {\text{I}}_{{\text{C}}} } \right)^{{{1 \mathord{\left/ {\vphantom {1 2}} \right. \kern-\nulldelimiterspace} 2}}} }}{{\sigma {\text{h}}^{3} }}$$

7 $${\text{Q}}_{{{\text{vib}}}} = \mathop \prod \limits_{{\text{i}}}\frac{{{\text{e}}^{{ - {\text{u}}_{{{{_{{\text{i}}}} \mathord{\left/ {\vphantom {{\text{i}} 2}} \right. \kern-\nulldelimiterspace} 2}}} }} }}{{1-{\text{e}}^{{ - {\text{u}}_{_{\text{i}}} }} }}$$

where V is the volume, M is the mass, I_A_ is the moment of inertia around the axis A of rotation, σ is the symmetry number of the molecule, and u_i_ = hcω_i_/kT, where h represents the Planck constant, k is the Boltzmann constant, T is the temperature in degrees Kelvin, c is the speed of light, and ω is the harmonic frequency in cm^−1^. The Teller-Redlich [62] approximation is done as follows:

8 $$\left( {\frac{{\text{M}}}{{\text{M}}^{\prime}}} \right)_{{{\text{AX}}}}^{3/2} \left( {\frac{{{\text{I}}_{{\text{A}}} {\text{I}}_{{\text{B}}} {\text{I}}_{{\text{C}}} }}{{{{\text{I}}^{\prime}}_{{\text{A}}} {{\text{I}}^{\prime}}_{{\text{B}}} {{\text{I}}^{\prime}}_{{\text{C}}}}}} \right)_{{{\text{AX}}}}^{1/2} \left( {\frac{{{\text{m}}^{\prime}}}{{\text{m}}}} \right)_{{\text{X}}}^{{3{\text{n}}/2}} = \mathop \prod \limits_{{\text{i}}} \frac{{{\text{u}}_{_{{\text{i}}}} }}{{{{\text{u}}^{\prime}}_{_{{\text{i}}}} }}$$

Therefore, the ratio of the translational partition function and the ratio of the rotational partition function can be expressed by the vibrational frequency. Through algorism transformation, the reduced partition function ratio (RPFR(AX/AX^’^)) is defined as follows:

9 $${\text{RPFR}}({\text{AX}}/{\text{AX}}^{\prime}) = \frac{\sigma }{{\sigma}^{\prime}}{\text{K}} = \mathop \prod \limits_{}^{{3{\text{n}} - 6}} \frac{{\text{u}}}{{{\text{u}}}^{\prime}}\left( {\frac{{{\text{e}}^{{ - \frac{{\text{u}}}{2}}} }}{{{\text{e}}^{{ - \frac{{{\text{u}}}^{\prime}}{2}}} }}} \right)\left( {\frac{{1 - {\text{e}}^{{ - {\text{u}}^{\prime}}} }}{{1 - {\text{e}}^{{ - {\text{u}}}}}}} \right)$$

When only one atom is to be exchanged in the molecular cluster, the RPFR is equivalent to the β factor [61], which is the isotopic fractionation factor between a compound and the ideal atomic gas [63]. Given the β factors of a pair of compounds or minerals in equilibrium, the isotope fractionation factor α can be derived from the ratio of their β factors: α = β_A_/β_B_, where A and B denote different compounds or minerals. The isotopic equilibrium fractionation between the two phases can then be obtained as follows:

10 $$\Delta_{{_{\text{A}} - _{\text{B}}}} \approx 10^{3} \ln {\upalpha }_{{{\text{A}} - {\text{B}}}} = 10^{3} ({\text{ln}}\beta_{{_{\text{A}}}} - {\text{ln}}\beta _{_{\text{B}}} )$$

## Results

### Influence of d_5×_, d_2×_, and d_1×_ on system electronic energy

**Fig. 6:**
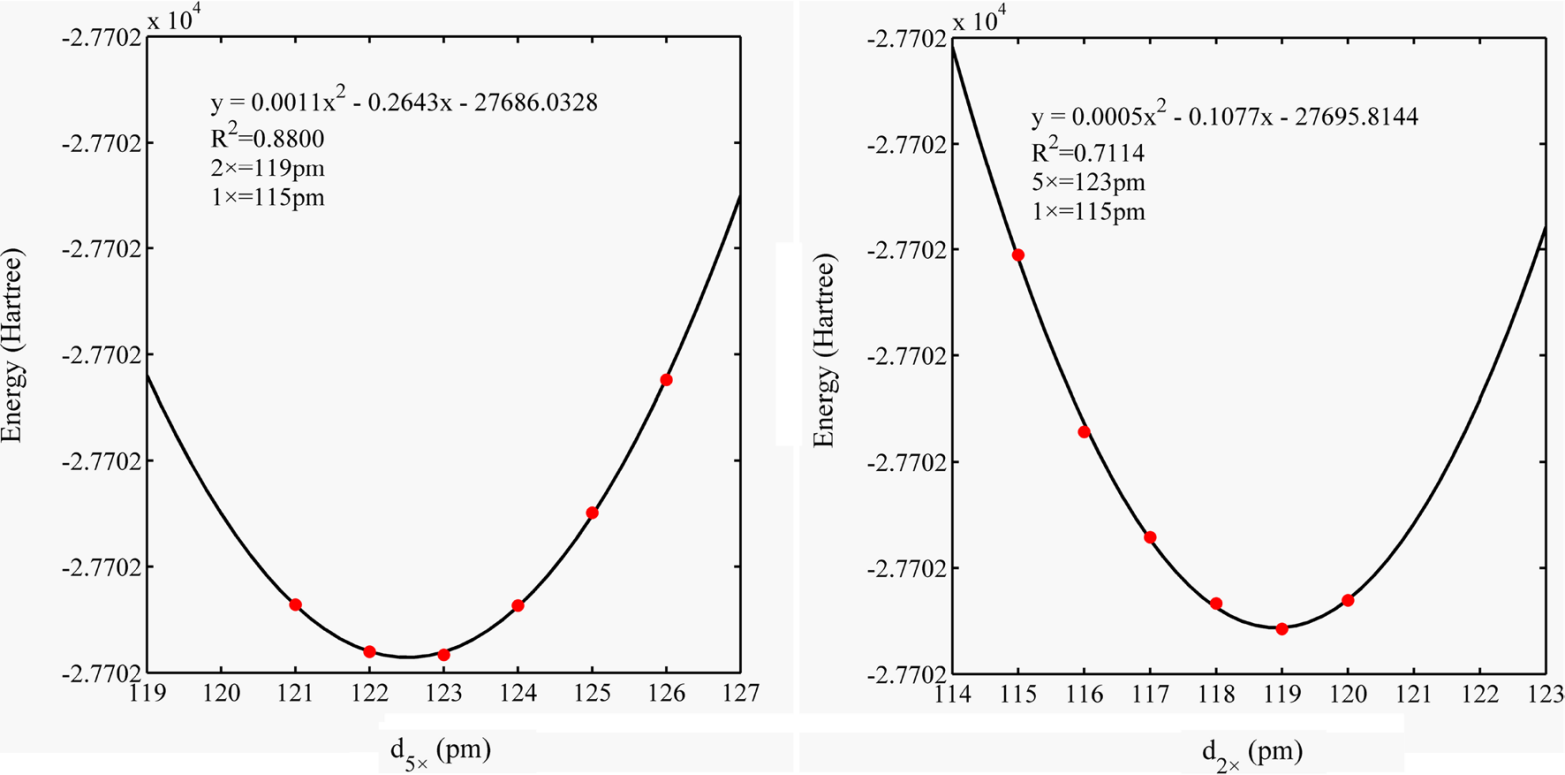
Electronic energy of the Cd–HAp system varies with d_5×_, d_2×_ and d_1×_

We collected the electronic energy values of the four systems and performed polynomial fitting. The results showed that the system electronic energy had a good parabolic relationship with d_5×_, d_2×_, and d_1×_, and the R^2^ value ranged from 0.60 to 0.99 (Figs. 3, 4, 5, 6). The PCA modes corresponding to the most stable configurations of the molecular clusters were 5 × 116, 5 × 116, 5 × 111/1 × 76, 5 × 123/2 × 122, and 5 × 123/2 × 119/1 × 115 pm for Si–Qtz, O–Qtz, Zn–Al LDH, Cd–Cal, and Cd–HAp, respectively.

### Effect of d_5×_, d_2×_, and d_1×_ on relative volume change

**Fig. 7:**
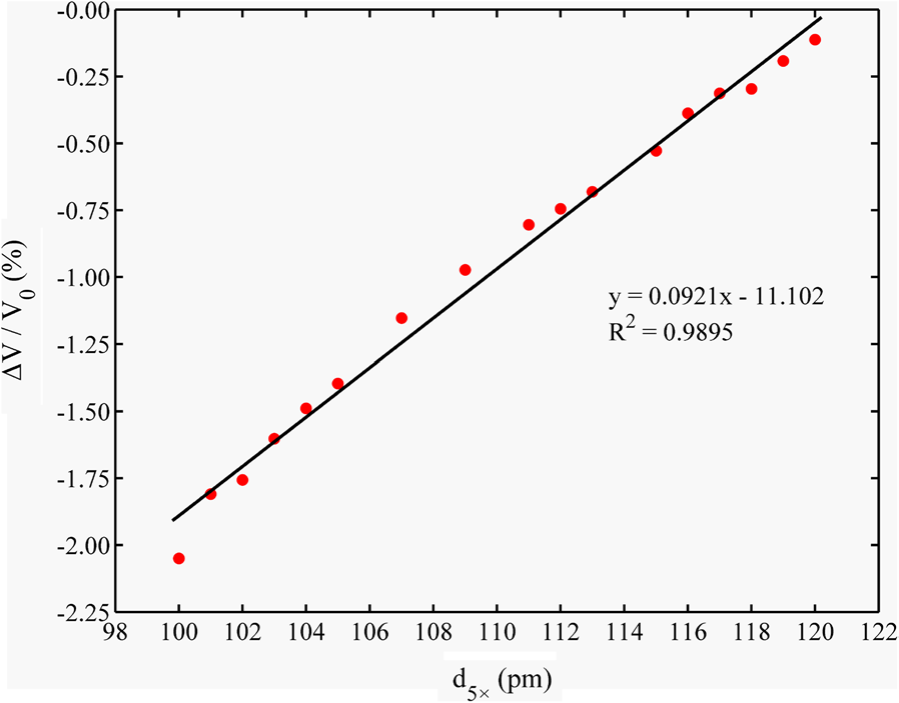
ΔV/V_0_ variations in the Si–Qtz molecular cluster

**Fig. 8:**
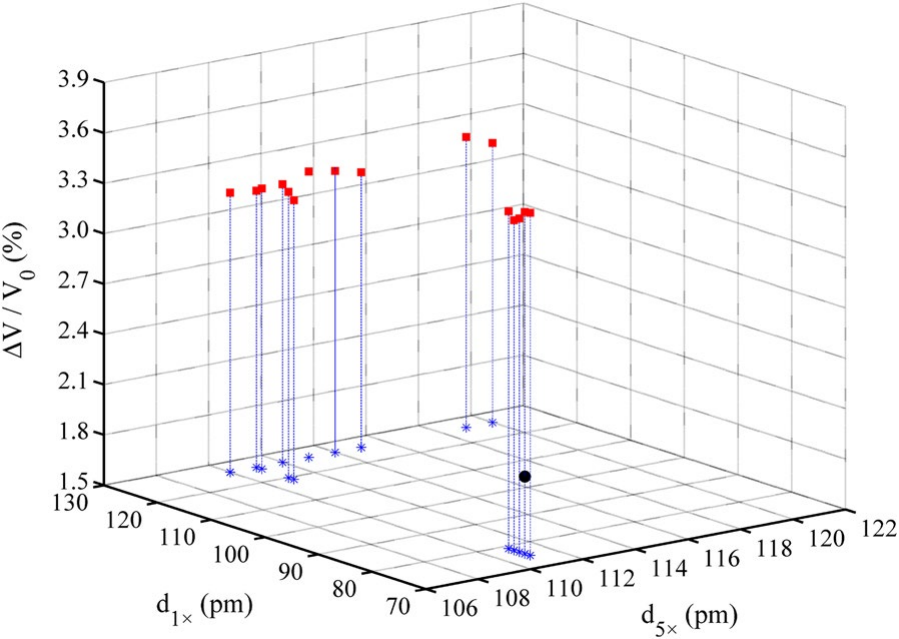
ΔV/V_0_ variations in the Zn–Al LDH molecular cluster. The red points were the relative volume changes for molecular cluster without interlayer anions, while the black point was that for molecular cluster with interlayer anions

**Fig. 9:**
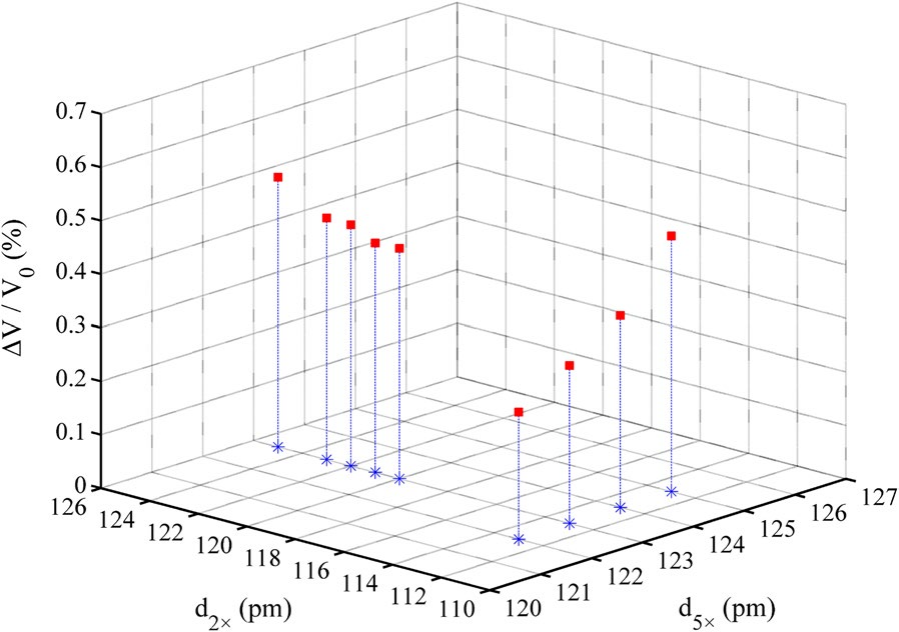
ΔV/V_0_ variations in the Cd–Cal molecular cluster

**Fig. 10:**
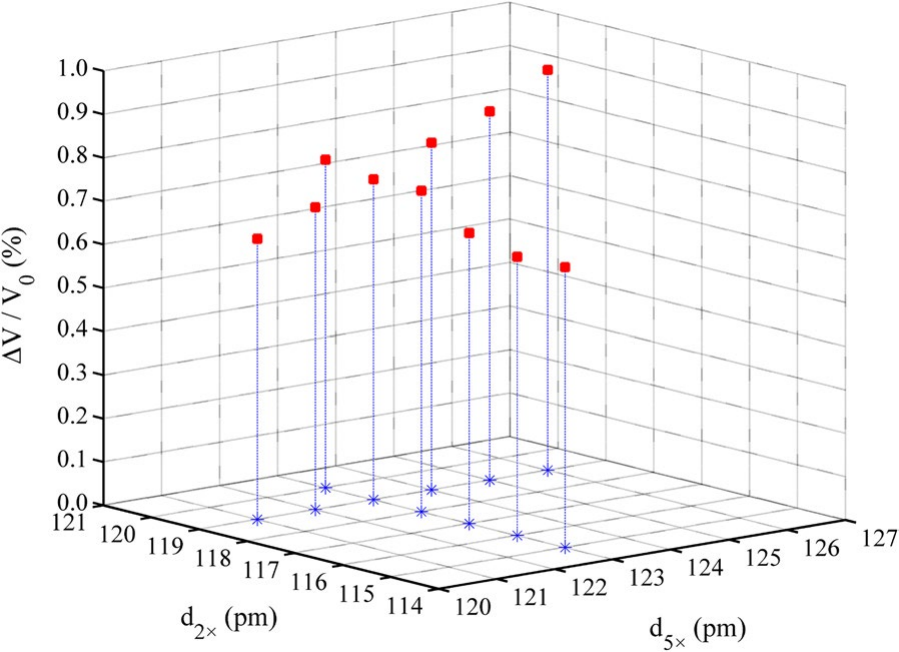
ΔV/V_0_ variations in the Cd–HAp molecular cluster

During the geometric optimization process, the volume of the Si–Qtz molecular cluster decreased, with ΔV/V_0_ ranging from − 2.05 to  − 0.11% (Fig. 7). We observed an extremely strong linear correlation between ΔV/V_0_ and d_5×_ (R^2^ = 0.9895). ΔV/V_0_ gradually increased with an increase in d_5×_. We recorded the ΔV/V_0_ value as  − 0.39% when the Si–Qtz molecular cluster reached the optimal structure. However, the volume of the Zn–Al LDH molecular cluster without interlayer anions expanded during the optimization process, with ΔV/V_0_ ranging from 3.17 to 3.54% (Fig. 8). The relative volume change was 3.54% when the system reached its lowest electronic energy configuration (5 × 111/1 × 76 pm). But when interlayer anions were introduced, the relative volume change reached 1.96% for the lowest electronic energy configuration (5 × 111/1 × 76 pm). As shown in Fig. 9, the volume of Cd–Cal expanded during the optimization process ranging from 0.24 to 0.50%. When the structure reached the maximum stability (5 × 123/2 × 122 pm), the volume expansion was 0.45% (Table 4). The volume of the Cd–HAp molecular clusters also expanded during the optimization process, with a volume expansion of 0.64–0.92%. We found that ΔV/V_0_ was 0.74% when the system reached the most stable configuration (5 × 123/2 × 119/1 × 115 pm) (Fig. 10), which is identical to the 0.7% reported by He et al. [25].

## Discussion

### Geometrically optimized configuration

In this study, we obtained an average Si–O bond length in the Qtz cluster with the lowest electronic energy of 1.63 Å (Table 3). Purton et al. [64] used ab initio Local Density Functional (LDF) theory to study the structure of α–quartz, and obtained an average Si–O bond length of 1.62 Å. He and Liu [22] calculated an average Si–O bond length of 1.61 Å on the 6–311G (2df) level. Hazen et al. [39] and Kihara [48] obtained the average Si–O bond lengths of 1.61 Å and 1.62 Å, respectively, using X-ray diffraction. Our results are consistent with those of previous theoretical calculations and experimental studies.

**Table 3:** Isotopic equilibrium fractionation between molecular clusters and their corresponding aqueous solutions

Cluster	Calculated bond length (Å)	Experimental bond length (Å)	1000lnβ (cluster, 298 K, ‰)	1000lnβ (aquocomplex, 298 K, ‰)	Δ _cluster-aquocomplex_ (298 K, ‰)
Si–Qtz	1.63, 1.62^a^, 1.61^b^	1.61^c^, 1.62^d^	73.65	71.63	2.02 (Si isotopes)
O–Qtz	1.63	1.61^c^	110.31	74.26	36.05 (O isotopes)
Zn–Al LDH	2.10	2.08^e^	4.87	3.75	1.12 (Zn isotopes)
Cd–Cal	2.28	2.30^f^	2.62	$2.88^{{\text{g}}}$	− 0.26 (Cd isotopes)

^a^Si–O bond length (α–quartz, Ab initio LDF theory, Purton et al. [64])

^b^Si–O bond length (quartz, DFT calculations, He and Liu [22])

^c^Si–O bond length (quartz, X-ray diffraction, Hazen et al. [39])

^d^Si–O bond length (quartz, X-ray diffraction at 848 K, Kihara [48])

^e^Zn–O bond length (X-ray diffraction and EXAFS, Gou et al. [3])

^f^Cd–O bond length (EXAFS, Bailey et al. [65])

^g^DFT calculations (He et al. [25])

The central Zn^2+^ in the Zn–Al LDH cluster had a coordination number of six, with an average Zn–O bond length of 2.10 Å. Gou et al. [3], by X-ray diffraction and EXAFS experiments, obtained an average Zn–O bond length of 2.08 Å (coordination number = 6.5) in the Zn–Al layer-like double hydroxides. Thus, the findings of these two studies are in good agreement with each other. For Cd–Cal, the average Cd–O (2.28 Å) bond length calculated in this study is consistent with the results obtained using EXAFS data (2.30 Å), as measured by Bailey et al. [65]. According to our procedure, the average Cd–O bond length in the Cd–HAp cluster was confirmed to be 2.39 Å, which is exactly the same as the average Cd–O (2.39 Å) bond length measured by Hata et al. [66] and the average Cd–O (2.38 Å) bond length calculated by He et al. [25].

### Relative volume changes

The relative volume change of mineral lattices during geometrical optimization has only been studied by a few researchers. Wang et al. [4] calculated the ΔV/V_0_ values of Ba-containing species by using the periodic boundary method. Except for Ba(OH)_2_(H_2_O)_8_, the relative volume change of which was -0.67%, the ΔV/V_0_ of the other species ranged from 1.26% to 6.30%, with an average of 4.46%. Ducher et al. [67] also used periodic boundary method to calculate the ΔV/V_0_ of various Zn-containing minerals (sulfide, carbonate, oxide, silicate, sulfate, and arsenate), which ranged between 1.40% and 5.80%, with an average of 3.56%. Thus, we concluded that for the majority of the studied systems, the ΔV/V_0_ values calculated using the molecular cluster method are much smaller (Table 4), approximately one-tenth of those obtained by the periodic boundary method.

**Table 4:** Relative volume change of solid molecular clusters

Cluster	ΔV/V_0_ (%)	
	This study	Previous studies
Si–Qtz	− 0.39	7.17^a^, − 2.66^b^, 4.61^c^, − 2.75^d^
Zn–Al LDH	1.96	–
Cd–Cal	0.45	3.54^e^, 3.47^f^
Cd–HAp	0.74	$0.7^{{\text{g}}}$, 3.43^ h^, 4.37^i^

^a^PBE [68]

^b^LDA [68]

^c^GGA(PW91) [69]

^d^LDA [69]

^e^CaCO_3_ [70]

^f^[71]

^g^DFT calculations [25]

^h^GGA–PBE [72]

^i^GGA–PW91 [73]

We found that the ΔV/V_0_ of quartz using the periodic boundary method calculated by the PBE and LDA functionals, primarily used by Méheut et al. [68], were 7.17% and -2.66%, respectively. Hamann [69] obtained the ΔV/V_0_ of quartz using GGA (4.61%, PW91) and LDA (-2.75%). The ΔV/V_0_ obtained in this study was -0.39%, which is substantially less than that obtained in previous studies. The Zn–Al LDH has a slightly larger expansion (1.96%) during geometric optimization, compared to that of other minerals in our study. But it is still smaller than those obtained by the periodic boundary method. The volume of the calculated Cd-containing calcite also increased (0.45%). However, the relative volume change of the Mn-containing calcite (3.54%) [70] and tetrahedral AO_4_^2−^ group (A = S, Cr, Se)-doped calcite (3.47%) [71] calculated by the periodic boundary method were larger than that obtained during this study. For HAp without impurities, Sailuam et al. [72] calculated a ΔV/V_0_ of 3.43% using GGA–PBE as the exchange–correlation functional. Bhat et al. [73] obtained ΔV/V_0_ (4.37%) using the exchange–correlation functional GGA–PW91. In contrast, the ΔV/V_0_ of Cd–HAp (Cd/(Cd + Ca) = 1/13) calculated in this study was 0.74%, which is much smaller than the previously reported values.

### $\Delta^{{{{30} \mathord{\left/ {\vphantom {{30} {28}}} \right. \kern-\nulldelimiterspace} {28}}}} {\text{Si}}_{{{\text{Qtz - H}}_{4} {\text{SiO}}_{4} }}$ and $\Delta^{{{{18} \mathord{\left/ {\vphantom {{18} {16}}} \right. \kern-\nulldelimiterspace} {16}}}} {\text{O}}_{{{\text{Qtz - }}\left( {{\text{H}}_{2} {\text{O}}} \right)_{{\text{n}}} }}$

With different exchange–correlation functionals and basis sets, 1000lnβ may be different. At 298 K, Méheut et al. [74] calculated the silicon β-factor of quartz as 69‰ and the oxygen β-factor as 102‰. At the same temperature, our silicon β–factor (73.65‰) and oxygen β-factors (110.31‰) were slightly larger than those reported by Méheut et al. [74]. However, when calculating the fractionation factor α, the difference in the β-factor for the same mineral can be offset. No substantial difference was observed between the $\Delta^{{{{18} \mathord{\left/ {\vphantom {{18} {16}}} \right. \kern-\nulldelimiterspace} {16}}}} {\text{O}}_{{{\text{quartz - }}\left( {{\text{H}}_{2} {\text{O}}} \right)}}$ value reported by Méheut et al. [68] and that obtained in this study (31.37‰ vs. 36.05‰). We compared the silicon and oxygen fractionation factors calculated in this study with previous experimental and theoretical data.

**Fig. 11:**
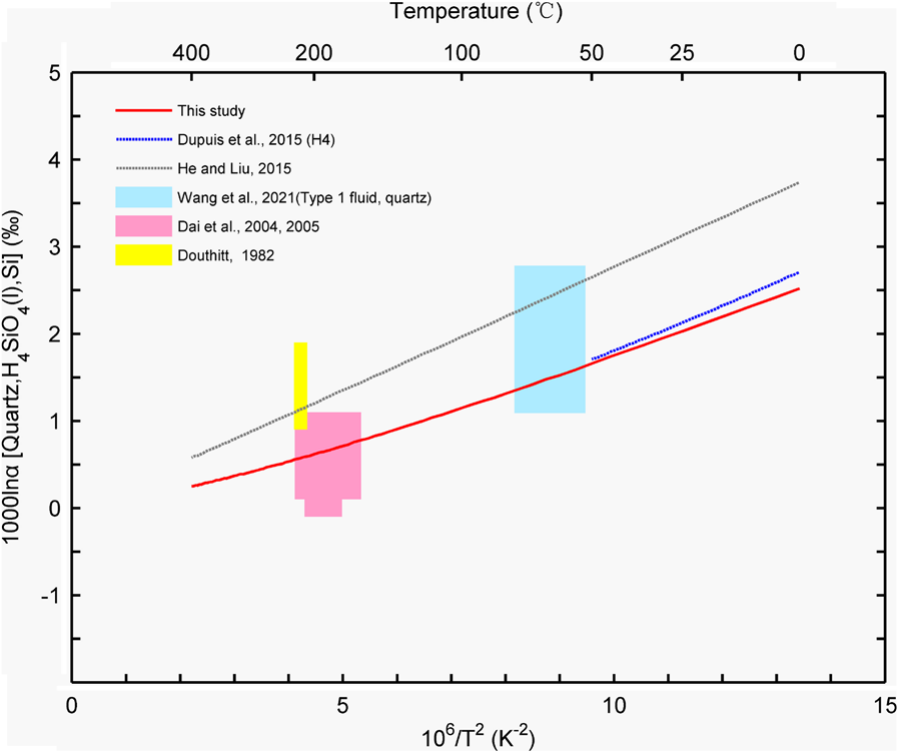
Theoretical Si isotope equilibrium fractionation factors between quartz and aqueous H_4_SiO_4_ solution [5, 7, 22, 75–82]

He and Liu [22] calculated the Si isotopic equilibrium fractionation between quartz and H_4_SiO_4_ solution at 273–673 K using B3LYP/6–311G(2df). They thought that a large Si isotopic equilibrium fractionation factor of approximately 3.3‰ exists between quartz and H_4_SiO_4_ solution at 298 K. Dupuis et al. [75] calculated the fractionation factors of Si isotopes between quartz and aqueous H_4_SiO_4_ at 273–323 K using first-principles methods, and found that at 300 K, the fractionation between quartz and H_4_SiO_4_ was + 2.1 ± 0.2‰. Figure 11 shows that our calculation results are in good agreement with those of Dupuis et al. [75]. Under neutral and acidic conditions, Si often exists in the form of aqueous H_4_SiO_4_ solutions. We considered the experimentally determined ∆^30/28^Si_quartz-aqueous solution_ to be $\Delta^{30/28}{\text{Si}}_{{\text {quartz-}}\text{H}_{4}{\text{SiO}}_{4}}$. From this perspective, the available experimental data [5, 7, 76, 77] match the existing theoretical data.

**Fig. 12:**
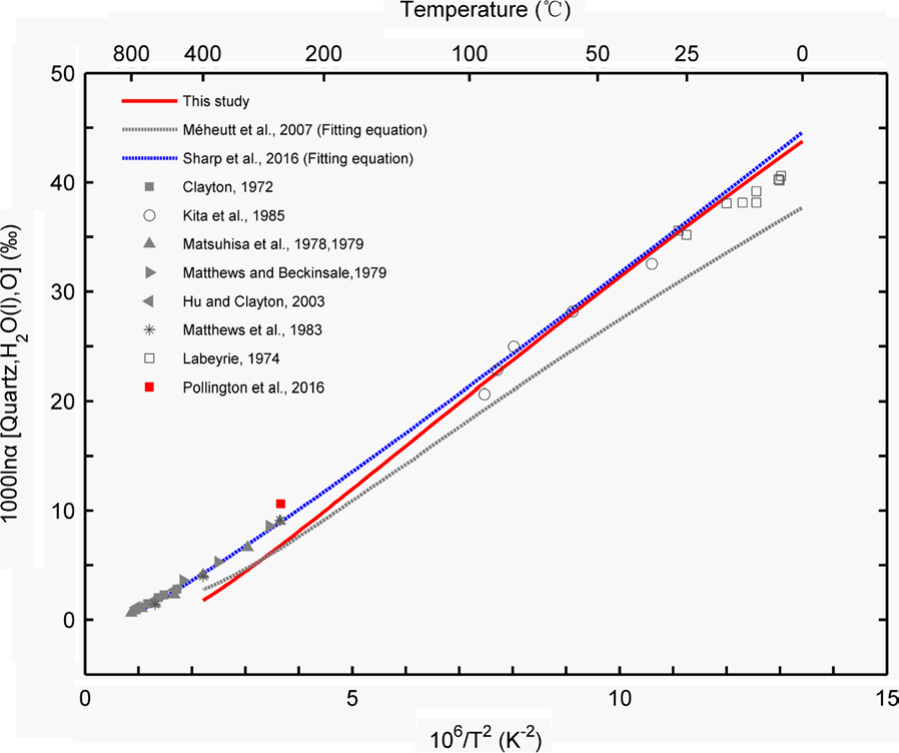
Theoretical O isotope equilibrium fractionation factors between quartz and liquid water [6, 68, 83–91]

To verify the reliability of the VVCM method, we further calculated the equilibrium oxygen isotope fractionation factor between quartz and the aqueous solution and then compared our result with previous experimental and theoretical results (Fig. 12). In the low-temperature range, compared to the theoretical results of the linear fitting reported by Méheut et al. [68], our calculated results are more consistent with the experimental values [6, 83] and the fitting equation based on experimental data [84]. However, at high temperatures, both theoretical results are less than the experimental value [85–91]. For example, at 673 K, our calculated oxygen isotope equilibrium fractionation factor and the experimental values reported by Méheut et al. [68] and Sharp et al. [84] were 1.79‰, 2.77‰, and 4.25‰, respectively.

In conclusion, the quartz geometric optimization and calculation of traditional/nontraditional stable isotope fractionation factors was accurate using the improved VVCM method. Finally, we can reliably apply this method to other systems such as Zn–Al LDH and Cd–Cal.

### $\Delta^{{{{66} \mathord{\left/ {\vphantom {{66} {64}}} \right. \kern-\nulldelimiterspace} {64}}}} {\text{Zn}}_{{{\text{Zn}} - {\text{Al LDH - Zn}}\left( {{\text{H}}_{2} {\text{O}}} \right)_{{\text{n}}}^{2 + } }}$ and $\Delta^{{{{114} \mathord{\left/ {\vphantom {{114} {110}}} \right. \kern-\nulldelimiterspace} {110}}}} {\text{Cd}}_{{\left( {{\text{Cd}} - {\text{Cal}}} \right) - {\text{Cd}}\left( {{\text{H}}_{2} {\text{O}}} \right)_{\text{n}}^{2 +}}}$

Because no experimental or theoretical data on Zn isotope fractionation between Zn–Al LDH and the solution have been published, we chose the Zn isotope fractionation produced during Zn^2+^ adsorption on the aluminum oxide/hydroxide surface for comparison. As reported by Komárek et al. [92] and Gou et al. [93], at higher surface coverage, Zn–Al LDH was detected by XRD on the surface of γ-Al_2_O_3_ with a fractionation factor of ∆^66/64^Zn_solid-aq_ = 0.02 ± 0.07‰. Pokrovsky et al. [94] also reported lower fractionation on corundum (∆^66/64^Zn_solid-aq_ = 0.19 ± 0.05‰) and gibbsite (∆^66/64^Zn_solid-aq_ = 0.10 ± 0.05‰) surfaces, suggesting that the potential formation of Zn–Al LDH on the surface of gibbsite and corundum is similar to that of γ-Al_2_O_3._ Although the size of calculated isotope fractionation (1.12‰) was larger than that obtained experimentally, the fractionation directions were consistent with each other. In fact, experimentally determined Zn isotope fractionation factor for adsorption on aluminum oxides/hydroxides may be complicated by mixed adsorption and precipitation. In future, we hope that Zn isotope fractionation can be experimentally determined for the individual Zn–Al LDH precipitation.

The calculated isotopic equilibrium fractionation between Cd–Cal and Cd-containing aqueous solution was -0.26‰. Previous studies on precipitated calcite from artificial seawater solutions reported the entry of Cd^2+^ into the calcite from seawater, with the Cd equilibrium isotope fractionation, ∆^114/110^Cd_calcite-seawater_ = -0.45 ± 0.12‰ [95]. Furthermore, Xie et al. [96] revealed apparent Cd isotope fractionation in the coprecipitation of Cd–calcite, with ∆^114/110^Cd _calcite-aqueous_ = -0.38 ± 0.18‰ to -0.20 ± 0.12‰. Our theoretical calculation results are consistent with previous experimental results.

## Conclusions

We used the first-principles calculation method to geometrically optimize the molecular clusters of Qtz, Zn–Al LDH, and Cd–Cal and obtained relative volume changes of -0.39%, 1.96%, and 0.45%, respectively. Compared to the periodic boundary method, the improved VVCM method slightly changes the molecular cluster volume during optimization, which increased the accuracy of our simulations. We also calculated the isotopic equilibrium fractionation between Qtz, Zn–Al LDH, Cd–Cal, and their corresponding aqueous solutions at 298 K, which were as follows: $\Delta^{{{{30} \mathord{\left/ {\vphantom {{30} {28}}} \right. \kern-\nulldelimiterspace} {28}}}} {\text{Si}}_{{{\text{Qtz - H}}_{4} {\text{SiO}}_{4} }} = 2.20{\permil}$, $\Delta^{{{{18} \mathord{\left/ {\vphantom {{18} {16}}} \right. \kern-\nulldelimiterspace} {16}}}} {\text{O}}_{{{\text{Qtz - }}\left( {{\text{H}}_{2} {\text{O}}} \right)_{{\text{n}}} }} = 36.05{\permil}$, $\Delta^{{{{66} \mathord{\left/ {\vphantom {{66} {64}}} \right. \kern-\nulldelimiterspace} {64}}}} {\text{Zn}}_{{{\text{Zn}} - {\text{Al LDH - Zn}}\left( {{\text{H}}_{2} {\text{O}}} \right)_{{\text{n}}}^{2 + } }} = 1.12{\permil}$and $\Delta^{{{{114} \mathord{\left/ {\vphantom {{114} {110}}} \right. \kern-\nulldelimiterspace} {110}}}} {\text{Cd}}_{{\left( {{\text{Cd}} - {\text{Cal}}} \right) - {\text{Cd}}\left( {{\text{H}}_{2} {\text{O}}} \right)_{{\text{n}}}^{2 + } }} = - 0.26{\permil}$. These results are consistent with those of previous studies, confirming the reliability of the improved VVCM method for crystal-lattice optimization.

## Supplementary Information

**Additional file 1: Figure S1**. Hydrogen bonds connected water molecules and scheme of point charge arrangements (PCA) for 1×, 3×, 4×, and 5×.

## Data Availability

The data sets that support the conclusions of this study are included in the study.

## Abbreviations

VVCM: Volume variable cluster model
DFT: Density Functional Theory
Si–Qtz: Quartz centered on silicon atoms
O–Qtz: Quartz centered on oxygen atoms
LDH: Layered double hydroxide
Cd–Cal: Cd–containing calcite
Cd–HAp: Cd–containing hydroxyapatite
PCA: Point charge arrangement
