# Supplementary material for: Improved volume variable cluster model method for crystal-lattice optimization: effect on isotope fractionation factor
Source: Geochem Trans. 2022 May 22;23:1. doi: 10.1186/s12932-022-00078-6 (PMC9124387; doi:10.1186/s12932-022-00078-6)
Supplement: Supplementary file 1 — Additional file 1: Figure S1. Hydrogen bonds connected water molecules and scheme of point charge arrangements (PCA) for 1×, 3×, 4×, and 5×. [file 12932_2022_78_MOESM1_ESM.docx]

**Additional file 1**

**Improved volume variable cluster model method for crystal-lattice optimization: Effect on isotope fractionation factor**

**Yan–Fang Wang^1^, Xin–Yue Ji^1^, Le–Cai Xing^1, 2^, Peng–Dong Wang^1^, Jian Liu^1^, Tian–Di Zhang^1^, Hao–Nan Zhao^1^, and Hong–Tao He^1, 2^**

^1^School of Earth Science and Engineering, Hebei University of Engineering, Handan 056038, China

^2^Key Laboratory of Resource Survey and Research of Hebei Province, Hebei University of Engineering, Handan 056038, China

*Geochemical Transactions*


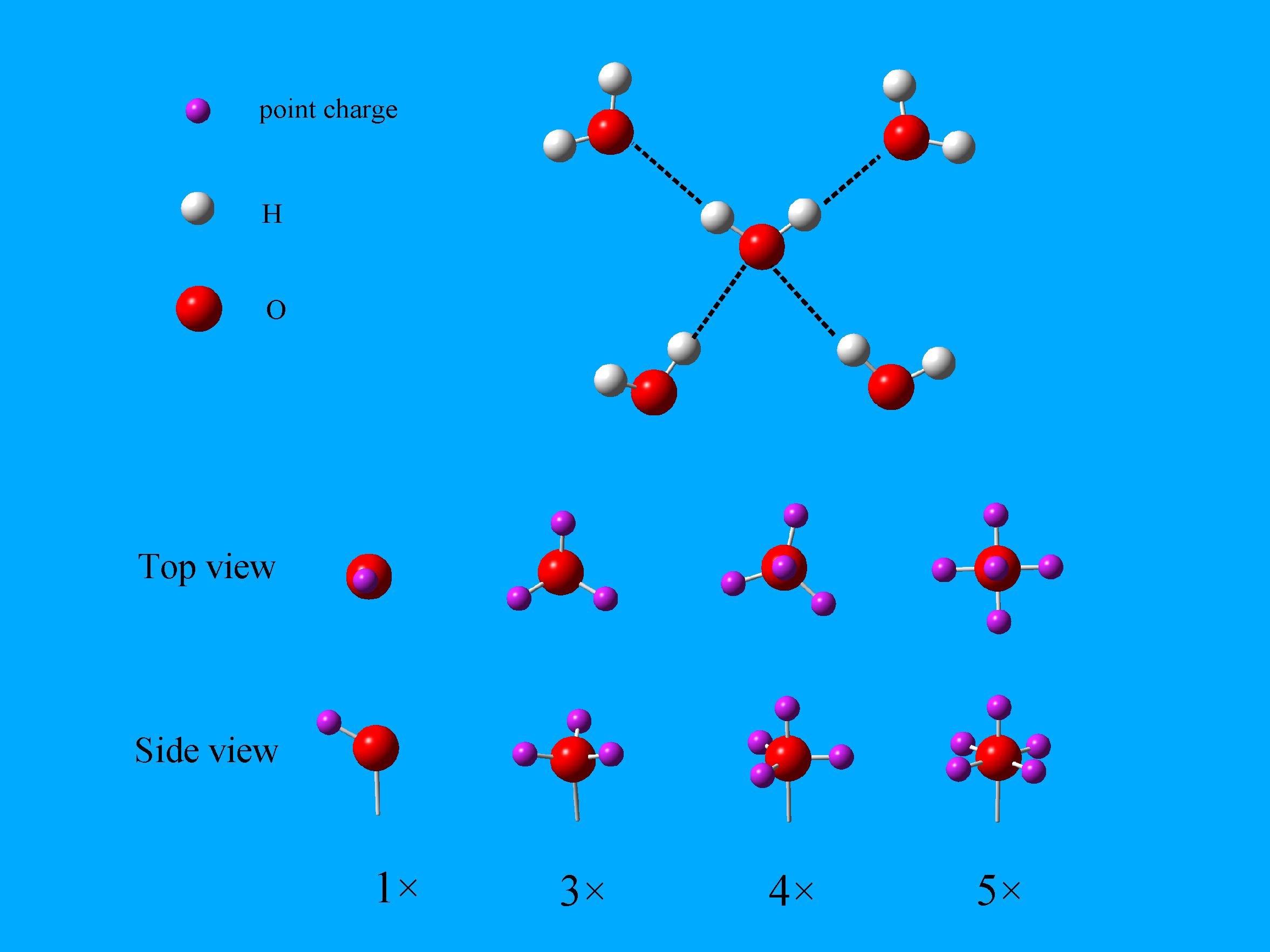


**Additional file 1: Fig. S1.** Hydrogen bonds connected water molecules and scheme of point charge arrangements (PCA) for 1×, 3×, 4×, and 5×
